# Dietary diversity and nutritional status of children attending early childhood development centres in Vhembe District, Limpopo province, South Africa

**DOI:** 10.1017/jns.2023.78

**Published:** 2023-08-11

**Authors:** Selekane Ananias Motadi, Mthokozisi Kwazi Zuma, Jeanne H. Freeland-Graves, Xikombiso Gertrude Mbhenyane

**Affiliations:** 1 Division of Human Nutrition, Faculty of Medicine and Health Sciences, Stellenbosch University, PO Box 241, Cape Town 8000, South Africa; 2 Department of Nutrition, Faculty of Health Sciences, University of Venda, Thohoyandou, South Africa; 3 Nutritional Sciences, University of Texas at Austin, Austin, TX, USA; 4 Smallholder Agricultural Development Unit, Agricultural Research Council, Pretoria 0002, South Africa

**Keywords:** Anthropometric status, Associations, Children, Dietary diversity, Malnutrition, AOR, adjusted odds ratio, CI, confidence interval, DD, dietary diversity score, ECD, early childhood development, sd, standard deviation, ZAR, South African Rand

## Abstract

The present study assessed dietary diversity and anthropometric status of children attending early development centres in South Africa. In the Vhembe District of Limpopo province, South Africa, 273 children were conveniently chosen from 8 randomly selected early childhood development centres for a cross-sectional study. Data were gathered via a questionnaire administered by the interviewer in June 2021. Height, body weight and mid-upper arm circumference were measured to assess anthropometric status. A 24-h dietary recall was obtained to provide information on dietary diversity. The prevalence of underweight, wasting and stunting was 9, 4 and 26 %, respectively. More than half of the children had a low dietary diversity score, according to the Food and Agriculture Organization scoring system for children. Grains, roots, tubers, dairy products, other fruits and vegetables, and flesh-based foods were the highest consumed food groups. The lowest consumption was for eggs, vitamin A-rich fruits and vegetables, legumes and nuts. Height for age and weight for age were significantly associated with dietary diversity score, but not weight for height. Children who did not meet the reference value of greater than 4 for dietary diversity had a significant risk of being underweight (AOR 0⋅25, 95 % CI 0⋅08, 0⋅75) and stunted (AOR 0⋅32, 95 % CI 0⋅14, 0⋅74). The nutritional status of the children was impacted by a lack of adequate dietary diversity. Young children in rural areas need to receive a wide range of food to promote greater diversification of diets in order to diminish the risk of undernutrition.

## Introduction

Populations living in most low- and middle-income countries experience public health issues related to nutrition because of their monotonous starchy- and cereal-based diets. This type of diet frequently contains little or no animal products, and few fruits and vegetables^(1–3)^. Nutrient deficiencies may occur when food is consumed in insufficient quantities and/or of low quality of both diversity and cleanliness. A diverse diet is crucial to ensure that children are receiving the vital nutrients needed^(4)^. The majority of children in the rural regions of northern South Africa are at risk for poor nutritional status and micronutrient deficiencies due to inadequate dietary intake of protein, energy and micronutrients, coupled with poor bioavailability^(5,6)^. According to the 2012 South African National Health and Nutrition Examination Survey, the prevalence of underweight, wasting and stunting in South Africa was 6⋅1, 3 and 22 %, respectively. In 2016, the South African Demographic Health Survey documented that 27 % of children under the age of five exhibited stunted growth.

Children under five years of age are the most vulnerable to adverse health consequences from inadequate diets and are at a stage where dietary habits are in development^(1)^. Shaping food consumption patterns in early childhood is essential, as these can affect future health^(7)^. Thus, it is critical to establish healthy food preferences in early childhood that will continue throughout the life span^(8)^. Although food preferences are essential, young children still rely on the meals that their parents or caregivers choose^(7,9,10)^.

In 1994, the South African government introduced meal plans in pre-schools in the rural regions to improve the nutritional adequacy of children's diets and the ability of children to learn^(11–13)^. One of the initiatives was to provide a government subsidy for the early childhood development (ECD) programmes. Subsequently, studies were conducted to assess dietary diversity of the general population and children^(3,14,15)^. These reported a high prevalence of low dietary diversity of 38⋅23 %^(15)^, 45 %^(14)^ and 61 %^(3)^ among children. One of these also reported that 29 % of children had stunting, 13 % had underweight and 6 % were wasted^(3)^. An amount of ZAR 15 (0⋅82 €) per day was given for 264 days/years per child to the preschools; this amount covered food, academic stationery and teacher salaries. Evaluations of the ECD programmes indicated problems with this provision of nutritional support^(16)^. This was especially true for those under the age of two, and for those who are living in poverty and underprivileged areas^(16,17)^. Some pre-schools did not receive the government subsidy for a particular year because application was on a yearly basis.These studies suggested that more research should be done on the relationship between dietary diversity score and nutritional status, as well as other factors associated with low dietary diversity among pre-school children. They also recommended that South Africa endeavour to ensure that all households have access to food, water and sanitation, and that dietary diversity can be utilised as a rapid and simple measure of the diet's sufficiency in micronutrients^(3,14,15)^. The aim of the present study is to investigate anthropometric status, and factors associated with low dietary diversity among children attending ECD centres in Musina Municipality in Vhembe District, Limpopo province, South Africa.

## Methods

### Study design and setting

A cross-sectional study was conducted to investigate dietary diversity and anthropometric status of children attending the ECD centres. A list of ECD centres was provided by the Vhembe District Department of Education. The research was conducted in the Musina local municipality in Vhembe District, one of the five municipal districts of Limpopo province. It was selected because of the continuous persistence of food insecurity, hunger and high unemployment rate of almost 24 %^(18,19)^. In this district, 70 % of population live under food poverty line, with incomes below ZAR 561⋅00 (28 €) per person per month^(19)^. According to Statistics South Africa, it has a population of 132 009, with 40 200 children between the age of 0–14 years^(18)^. Data were collected by the researcher (a registered nutritionist), two research assistants (Qualified Nutritionists) and eight field workers in June 2021. Field workers and research assistants received training on procedures and methods of data collection 2 weeks before data collection started by the researcher, promoter and a postdoctoral fellow. The appointment of fieldworkers was based on their expertise which were required for the study and for good general practice.

### Study population, sample size and sampling procedure

Children, aged 3–4 years, whose parents consented and were present on the day of data collection, were included in the study. Children with physical disabilities that made it challenging to conduct anthropometric measurements due to a lack of equipment to measure their height were excluded, such as those who had trouble standing on their own.

Eight pre-schools were selected by simple random sampling from the twenty-five pre-schools in Musina Municipality. The sample size was calculated using the Solvin's formula, using a population size of 4590 young children at 25 pre-schools. A 0⋅05 was considered a tolerable level of error and a 95 % confidence level. The formula yielded 368 subjects; an addition of 10 % was added for attrition. A total of 273 parents/caregivers consented for their children to participate and their children were selected.

### Data collection and variables measured

A separate classroom at each ECD centre was requested to be used to ensure privacy for the children and their parents/caregivers. Two stations were arranged; variables measured were socio-demographic characteristics, anthropometrics and dietary diversity at two separate stations.

The socio-demographic characteristics measured were child age, sex and information on government grants and parents/caregivers’ educational attainment, employment status, household income, housing type, access to water, type of energy used for cooking and type of toilet.

Anthropometric assessments were performed according to standard procedures of the International Society for the Advancement of Kinanthropometry^(20)^. Measurements were taken in duplicate, in children wearing light clothing without shoes, using calibrated equipment. Measures included standing height, weight and mid-upper arm circumference^(21)^. Height was measured to the nearest 0⋅1 cm using a portable stadiometer; and weight, to the nearest 0⋅01 kg on a portable Seca solar scale (Model 0213; Seca, Hammer Steindamm, Hamburg, Germany).

### Definitions of underweight, wasting and stunting

The anthropometric status of the children was categorised by using the appropriate cut-offs for classification established by the World Health Organization^(22)^. Measurements of weight and height were converted to age and sex-specific *z*-scores to establish anthropometric status, according to the World Health Organization Anthro and AnthroPlus^(23)^. For severe underweight, severe wasting and severe stunting, the cut-off marks were −3 sd. The cut-off values for weight-for-height were between +2 sd and +3 sd. Weight-for-height *z*-scores of between −2 and ≤−1 were used to define children at risk of wasting. When using weight for height, the *z*-score of above 1 sd was considered possible risk of overweight.

Dietary diversity was derived from data collected from a 24-h dietary recall. Information about all foods and beverages consumed during the previous 24 h was obtained by a one-day dietary recall administered by the researcher and fieldworkers, using a multiple pass method^(24)^. Parents/caregivers of the children were asked to visit the pre-schools on the day the data were collected and were requested to recall all foods that the child had consumed during the previous 24 h. Food cards were utilised to assist memories of the food items and quantities fed to the children. Using this 24-h diet recall, dietary diversity scores (DDs) were calculated as per the Food and Agriculture Organization guidelines^(25,26)^. The score was defined as the number of food groups consumed by the child during the previous 24 h. The dietary diversity score was based on the following seven food groups: grains, roots and tubers; legumes and nuts; dairy products (milk, yoghurt and cheese); flesh foods (meat, fish, poultry and liver/organ meats); eggs; vitamin A-rich fruits and vegetables; and other fruits and vegetables. The score ranged from 0 to 7. A score < 4 was considered to be low dietary diversity; whereas a score ≥ 4 was deemed adequate^(27)^. The researcher and field workers asked the cooks at each ECD centre about the food they fed the children the previous day and received copies of the menu in each ECD. The information obtained from the cooks at the ECD centre about the food consumed was added to the 24-h dietary recall.

### Ethical considerations

This study received ethical clearance from the Stellenbosch University Health Research Ethics Committee (S18/10/216), and approval from the provincial and district Department of Education in Vhembe. The Declaration of Helsinki^(28)^, good clinical practices and South African law were followed during all protocols. Prior to the study, parents of participants received both an oral and written description of the study including potential risks. All parents/caregivers of the children provided signed consent.

### Statistical analysis

Quantitative data were analysed using the Statistical Package for the Social Sciences (SPSS for Windows version 27, SPSS Inc., Chicago, IL, USA). A *χ*^2^ test was used to compare associations between dietary diversity score and socio-demographic characteristics. The number of food groups the child consumed the day before the survey was used to create the continuous variable for the diet diversity score. Each anthropometric result was given its own model, with DDs acting as the independent variable. The score of ≥4 was used as the reference category indicator.

To assess the association of dietary diversity on children who could be at a higher risk of having poor nutritional status. Lasso regression was used to screen important variables to be included in the models^(29)^. Other variables selected which were not significant in the final estimation step were excluded from the model. Even though Lasso did not select the dietary diversity score, it was included to determine if it affected nutritional status. The ratio of sample size to the number of variables exceed 20 for all the models considered. Bivariate model was conducted to identify association between the outcomes (weight for age and height for age) and independent (dietary diversity) variable. Multivariate models were created adjusting for child age, sex, parent or caregiver's marital status, level of education, employment, monthly household income, housing type, availability to water and toilet type. Models were run for the entire sample of children and for the age subgroups of children (3 and 4 years) to assess the influence of dietary diversity on children who may have a higher risk of poor nutritional status. The significance level was set at *P* < 0⋅05. The unadjusted odds ratio (OR) and the adjusted odds ratio (AOR) were calculated using a 95 % confidence interval (CI).

## Results

### Socio demographic characteristics and dietary diversity

Table 1 presents the socio-demographic characteristics of children and their parents/caregivers, according to the dietary diversity score. Most of the families were still using firewood for cooking and 56 % had low diet diversity. About 40⋅4 % of the children whose parents were unemployed exhibited low dietary diversity, as compared to 16⋅8 % of children whose parents were employed. About 30⋅7 % of children aged 3 years had low dietary diversity compared to 26⋅5 % of 4 year olds. Positive associations existed between dietary diversity and employment status (*P* = 0⋅03), household income (*P* = 0⋅04), access to water (*P* = 0⋅04) and type of toilet (*P* = 0⋅04). Of the children who received a government child grant, 51⋅6 % had a low dietary diversity score.

**Table 1.** Socio-demographic characteristics and dietary diversity (*n* 273)

Demographic	Dietary diversity score				*P*-value
	Low (<4) *n* %		Adequate (≥4) *n* %		
Parents/caregiver					
Educational level					
None	1	0⋅4	2	0⋅8	0⋅34
Low literacy^a^	12	4⋅4	8	2⋅9	
High literacy^b^	143	52⋅3	107	39⋅2	
Employment status					
Employed	46	16⋅8	30	11	**0**⋅**03**
Unemployed	110	40⋅4	87	31⋅8	
Household income (ZAR)^c^					
<2000	104	38⋅1	78	28⋅5	**0**⋅**04**
≥2000	52	19	39	14⋅4	
Housing					
Traditional house^d^	35	12⋅8	24	8⋅8	0⋅08
Shack^e^	32	11⋅7	21	7⋅8	
Brick and mortar	89	32⋅6	72	26⋅3	
Access to water					
River	7	2⋅6	13	4⋅7	**0**⋅**04**
Borehole^f^	31	11⋅4	12	4⋅4	
Communal tap	118	43⋅2	92	33⋅7	
Type of energy used for cooking					
Firewood	153	56	28	10⋅2	0⋅72
Gas	2	0⋅8	1	0⋅4	
Electricity	1	0⋅4	88	32⋅2	
Type of toilet					
Bush	14	5	6	2⋅2	**0**⋅**04**
Pit toilet	141	51⋅6	109	40	
Flushing toilet	1	0⋅4	2	0⋅8	
Child					
Age					
3 years	84	30⋅7	52	19	0⋅09
4 years	72	26⋅5	65	23⋅8	
Sex					
Boys	79	28⋅9	57	20⋅9	0⋅66
Girls	77	28⋅3	60	21⋅9	
Received grant					
Yes	141	51⋅6	109	40	0⋅43
No	14	5	9	3⋅4	

a Primary school.

b Secondary and tertiary level

c 1 euro is equivalent to R17⋅50.

d Build using mud.

e Corrugated iron.

f Composition not treated or altered.

### Anthropometric status of children

**Fig. 1.**
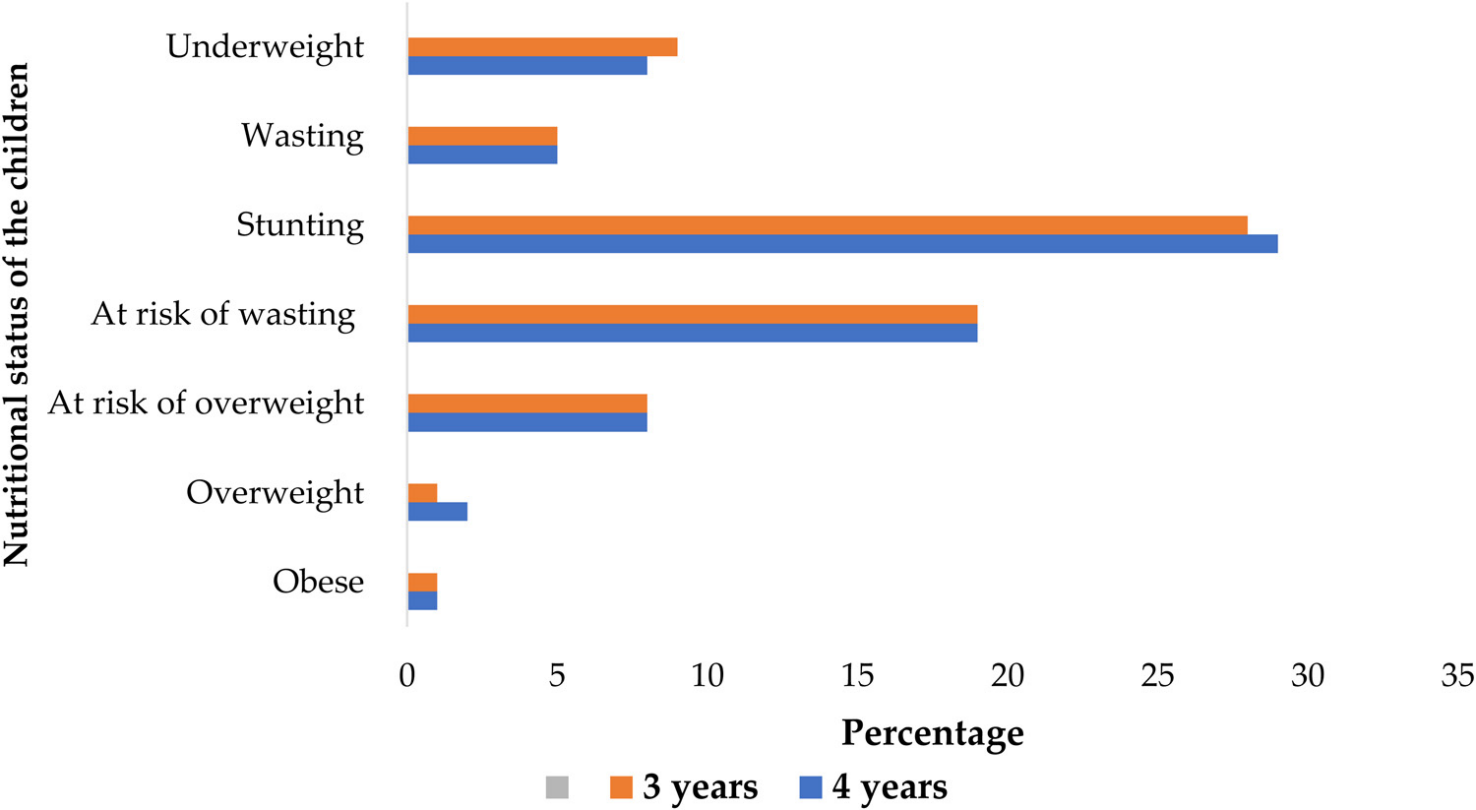
Anthropometric status of children in ECDs of Musina Municipality (*n* 273).

The anthropometric status of the children is illustrated in Fig. 1. The prevalence of underweight, wasting and stunting was 9, 4 and 26 %, respectively. The rate of underweight children was greater in 3-year-olds, while stunting was higher in 4-year-olds. Stunting appears to be a greater public health concern, with the proportion higher in the 4 years old. In contrast, overweight and obesity were minimal in these children. When using mid-upper arm circumference *z*-scores cut-offs of between −2⋅0 and −2⋅9, 3⋅9 % had moderate Undernutrition^30)^ (Fig. 1).

### Frequency of consumption of food groups

**Fig. 2.**
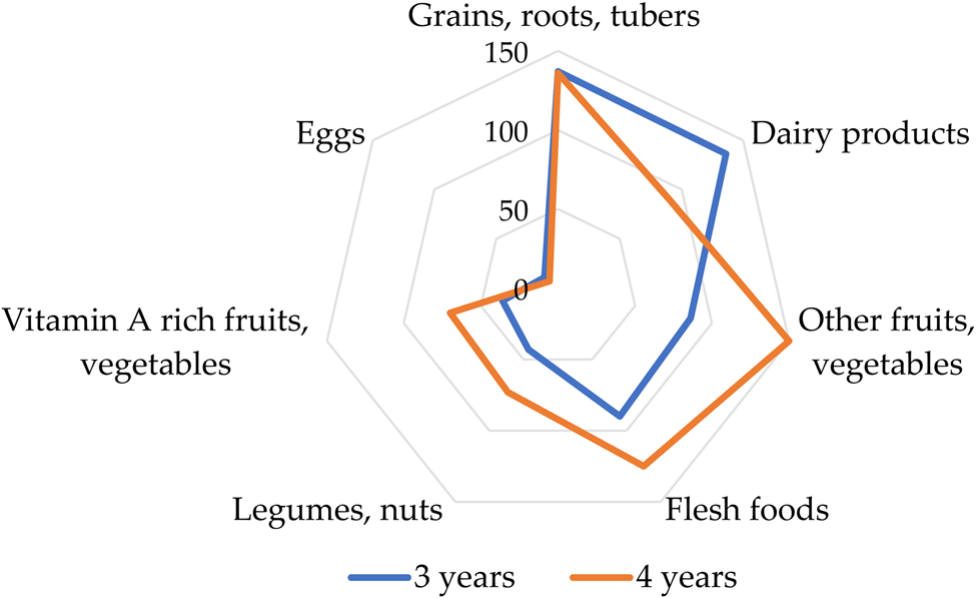
A spider plot of the frequency of consumption of food groups (*n* 273).

The frequency of consumption of the food groups is illustrated as a spider plot in Fig. 2. Food groups with the highest rates of consumption were grains, roots, tubers; dairy products; other fruits and vegetables; and flesh foods. The consumption of dairy products (*P* = 0⋅024), flesh foods (*P* = 0⋅012), other fruits and vegetables (*P* = 0⋅032) differed significantly between the age groups. The least consumed food groups were eggs; vitamin A-rich fruits and vegetables; legumes, and nuts. In 4-year-olds, dairy products were the least frequently consumed. The 3-year-old children consumed less vitamin A-rich fruits, vegetables, flesh food, legumes and nuts than the older children. An interesting finding in this study was the low dietary intake of foods high in vitamin A, eggs and legumes and nuts (Fig. 2).

### Dietary diversity scores of children

More than half (56 %) of the children had a low dietary diversity score compared to the 42 % who had adequate scores. When comparing children within their age group, 3-year-olds (62 %) exhibited a low dietary diversity as compared to 4-year-olds (52 %).

### Associations between anthropometric status and dietary diversity

Height for age and weight for age were positively associated with DDs. But no association was seen with weight for height (Table 2).

**Table 2.** Associations between anthropometric status and dietary diversity

Anthropometric status	Dietary diversity score				*P*-value
	Low *n* %		Adequate *n* %		
Weight for age					
Underweight (<−2 sd)	25	9⋅2	0	0	**0**⋅**02**
Normal	130	47⋅6	118	43⋅2	
Height for age					
Stunting (<−2 sd)	64	23⋅4	8	2⋅9	**0**⋅**01**
Normal	91	33⋅3	110	40⋅4	
Weight for height					
Wasting (<−2 sd)	12	4⋅4	0	0	0⋅29
Normal	143	52⋅4	118	43⋅2	

The weight-for-height *z*-score classification cut-off point between >+1 sd and <+2 sd was defined as at risk of overweight.

Table 3 shows the logistic regression analysis of associations between dietary diversity and anthropometric status of the children. Consumption of a low diversity diet was positively associated with weight for age and height for age but the same was not observed with weight for height. As the number of food categories declined, the likelihood of developing weight for age and height for age problems increased. Using a reference value of greater than 4, children who had a dietary diversity score of less than 4 had a significant risk of being underweight (AOR 0⋅25, 95 % CI 0⋅08, 0⋅75) and stunted (AOR 0⋅32, 95 % CI 0⋅14, 0⋅74), as compared to those whose dietary diversity score ≥ 4. Weight for height was not significantly associated with a reference value greater than 4 (unadjusted; OR 0⋅65, 95 % CI 0⋅43, 1⋅19; AOR 0⋅33, 95 % CI 0⋅32, 1⋅13) (Table 3).

**Table 3.** Bivariate and multivariate logistic regression analysis of the associations between dietary diversity score and nutritional status

		Bivariate	*P*-value	Multivariate	*P*-value
**Weight for age (<−2 sd)**					
Dietary diversity score	*n*	OR (95 % CI)		AOR (95 % CI)	
	36	0⋅34 (0⋅15, 1⋅76)	0⋅44	0⋅31 (0⋅13, 0⋅7)	0⋅02
3	119	0⋅91 (0⋅5, 1⋅54)	0⋅28	0⋅49 (0⋅26, 0⋅81)	0⋅01
4	39	0⋅76 (0⋅14, 3⋅81)	0⋅74	0⋅99 (0⋅94, 1⋅05)	0⋅62
5	57	0⋅46 (0⋅08, 2⋅23)	0⋅33	0⋅33 (0⋅09, 1⋅19)	0⋅26
6	22	1⋅32 (0⋅23, 6⋅86)	0⋅73	0⋅23 (0⋅52, 1⋅62)	0⋅76
Minimum dietary diversity < 4 (Food groups)		0⋅53 (0⋅29, 0⋅18)	0⋅38	0⋅25 (0⋅08, 0⋅75)	0⋅03
**Height for age (<−2 sd)**					
Dietary diversity score	*n*				
2	36	0⋅35 (0⋅15, 0⋅32)	0⋅02	0⋅22 (0⋅13, 0⋅76)	0⋅01
3	119	0⋅34 (0⋅33, 1⋅78)	0⋅45	0⋅29 (0⋅01, 0⋅84)	0⋅04
4	39	1⋅07 (0⋅31, 3⋅61)	0⋅91	0⋅22 (0⋅31, 0⋅64)	0⋅05
5	57	1⋅49 (0⋅53, 4⋅40)	0⋅46	0⋅57 (0⋅31, 1⋅05)	0⋅11
6	22	2⋅97 (0⋅85, 10⋅68)	0⋅09	0⋅16 (0⋅19, 1⋅38)	0⋅49
Minimum dietary diversity < 4 (Food groups)		0⋅69 (0⋅35, 1⋅38)	0⋅53	0⋅32 (0⋅14, 0⋅74)	0⋅01
**Weight for height (<−2 sd)**					
Dietary diversity score	*n*				
2	36	0. 54 (0⋅23, 1⋅32)	0⋅15	0⋅22 (0⋅16, 1⋅16)	0⋅19
3	119	0⋅85 (0⋅67, 1⋅08)	0⋅21	0⋅82 (0⋅64, 1⋅06)	0⋅13
4	39	1⋅19 (0⋅52, 2⋅76)	0⋅67	0⋅72 (0⋅44, 1⋅18)	0⋅26
5	57	0⋅96 (0⋅80, 1⋅21)	0⋅17	0⋅87 (0⋅68, 1⋅14)	0⋅12
6	22	0⋅98 (0⋅43, 2⋅36)	0⋅97	0⋅35 (0⋅64, 1⋅43)	0⋅34
Minimum dietary diversity < 4 (Food groups)		0⋅65 (0⋅43, 1⋅19)	0⋅68	0⋅33 (0⋅32, 1⋅13)	0⋅09

*P* < 0⋅05; adjusted for age, sex, income, marital status and employment CI confidence interval, OR (odds ratio) and AOR (adjusted odds ratio), the score of ≥4 was used as a reference category.

## Discussion

In the South African Musina municipality of the Vhembe District, low dietary diversity, stunting and underweight among pre-school children are problems. The study findings suggest that factors for poor dietary diversity were related to unemployment, low household income, water access and type of toilet. The current findings of poor dietary diversity are similar to others reported in developing countries in Africa such as Ethiopia^(31)^, Ghana^(32)^, Burkina Faso^(33)^ and the Northwest province in South Africa^(3)^. These values are lower than those observed in Tanzania by Khamis *et al.* in 2019^(34)^ . The diets of children in this investigation consisted primarily of carbohydrate-rich foods, vegetables and seasonal fruits, with only a few animal products. The lack of variety in these meals reflects their monotonous nature and has been linked directly to the insufficient intake of the nutrients needed for rapid growth of children^(34–36)^. Such an inadequate diet is concerning as it can increase susceptibility to infections and ailments, ultimately, leading to malnutrition^(37)^. Furthermore, a diverse diet in children also is a predictor of nutritional quality and density^(34,38,39)^ with low dietary diversity being positively associated with underweight and stunting. This relationship corresponds to the low DDs related to the underweight and stunting observed in the younger age group.

Multiple studies have demonstrated that poverty, low income and poor dietary diversity are the primary drivers of inadequate nutrition and malnutrition in rural populations^(40,41)^. This is true particularly in those that rely on social subsidies from the government with incomes below ZAR 2000⋅00 (112 euro) per month. Despite South Africa being a food secure country^(19)^, food insecurity and hunger are persistent in certain populations. Approximately 70 % of population lives under the food poverty line [incomes below R561⋅00 (31⋅66 euro)]. This income level is considered insufficient to purchase the variety of food needed for a nutritionally adequate diet of a child. It may explain why more than half of the children exhibited low dietary diversity. Interventions are needed to raise income, production of local nutritious foods and the level of education of parents and other caregivers^(42)^.

The effects of poor quality of diets in children are severe and may affect linear growth, cognition and muscle development. These conditions may be irreversible during childhood^(43)^. Furthermore, inadequate nutrition may limit educational attainment^(44,45)^ and adult functioning^(46)^. The lack of an association between dietary diversity and wasting in the present study may suggest that eating a variety of foods might not be the only cause of wasting in these children. Illness, infectious diseases and a lack of access to clean water are other factors that may impact wasting and stunting^(47,48)^. These findings are consistent with those of preceding reports by Khamis *et al.*^(34)^ and Modjadji *et al.*^(3)^ who found no association between dietary diversity and wasting. It is also plausible that wasting might be caused by short-term episodes of inadequate feeding or illness^(34,49)^, rather than the low dietary diversity observed here.

Insufficient intake of foods rich in vitamin A, legumes and nuts foods was not expected, as the Limpopo province is considered a bread and fruit basket of South Africa^(50,51)^. This area produces up to 60 % of all fruit, vegetables, maize meal and wheat. In fact, the province produces about 75 % of the mangoes, 65 % of papayas, 25 % of citrus, bananas and litchis, 60 % of avocados, 60 % of tomatoes, 19 % of potatoes and 35 % of oranges^(50,51)^ for the country. The lack of these foods in the diet suggests that families may sell produce to raise money to buy basic needs, rather than utilising these foods for their own consumption. These families do not appear to be following the South African Food-Based Dietary Guidelines that recommend that children should consume a variety of fresh fruits and vegetables (five servings) every day, as well as legumes on a regular basis^(52)^. Reasons could be lack of knowledge, inadequate money or spending on non-food items such as tobacco. Despite the increased awareness of the influence of fruits and vegetable consumption on health, low intakes remain a problem in low and middle countries, including South Africa^(53)^.

One element of a poor standard of living circumstance is an unimproved toilet^(54)^. So, it is not surprising that the present research observed an association between toilet type and dietary diversity. These results are consistent with an investigation conducted in Uganda by Rukundo *et al.*^(55)^ concerning toilet ownership and diversity scores. The lack of a toilet was related to being a household with food insecurity. Sanitary toilet facility is a measure to prevent diseases and improve the health condition of household members and is an indicator that determines the sanitation status of households. Children from households with food insecurity are more likely to have an insufficient variety of foods in their diets. The results of the present research reaffirm the necessity for food and nutrition security programmes that are mindful of housing, water, sanitation and hygiene challenges.

The present study is the first to report on the associations between household water access, sanitation and dietary diversity in South Africa. Villages in this rural area of the municipality still use communal taps that are used by all community members. Households may be forced to spend money that would have been used to buy food on water if the tap water is located too far away^(56)^. In water-stressed environments, fresh fruits and vegetables may be avoided in favour of cheaper foods which require less water to process and produce^(57)^. This finding is in consistent with those of investigations conducted in Ethiopia^(58)^, India^(57)^ and China^(56)^. These investigations reported that inadequate water availability was linked to lower odds of children achieving a diverse diet, which was reflected primarily by decreased odds of eating fruits and vegetables. Foods that need a lot of water to produce and prepare, such as fish, fruits and vegetables, are more likely to be found in households with better access to water^(56,57)^.

### Policy implications

At present, the South African government is trying to address nutrition-related problems by a variety of methods. These include nutrition education, food supplementation, National School Nutrition Program, micronutrient supplementation at health facilities, food fortification, diversification and utilisation of food-based dietary guidelines^(59–61)^. In addition, South Africa started creation of vegetables gardens at schools in order to improve children's healthy eating, household food security and create jobs for the community^(62)^. However, the practice of vegetable gardens is dwindling, and many are no longer in operation. Despite these efforts, poor dietary diversity in the Musina Municipality remains a problem.

Five change levers could improve dietary diversity and nutritional status of young children and be sustainable in rural Vhembe villages. These include (1) inclusion of locally available foods such as indigenous products into the pre-school menus and (2) the addition and emphasis on healthy eating into the learner's curriculum. At present, nutrition is included as only one of many topics forming part of the Life Orientation syllabus in South Africa^(63,64)^. Other levers are to (3) encourage the establishment of home and school vegetable gardens; (4) improve the current unemployment rate to generate higher household incomes to purchase food and (5) intensify and raise public awareness about the importance of dietary diversity. Finally, community health professionals could identify childhood malnutrition at an early stage by performing targeted screenings of children in remote regions.

### Strength and limitations

The sample population in this study was restricted to only one municipality in the Vhembe District. Thus, the findings cannot be generalised to all pre-schoolers in South Africa, but likely to prevail in similar environments. Additionally, the mother and caregivers’ estimates of the child's nutritional intake were obtained by 24-h dietary recall. This method may not accurately reflect consumption on a regular basis or include food consumed while at the pre-school; furthermore, it is also subject to recall bias. Moreover, there were no specific measurements of chronic family food insecurity, infection and disease and other possible contributing factors to dietary diversity and malnutrition. Finally, the study was conducted in winter; the nutritionally availability of most fruits and vegetables might be low as compared to the summer. Nonetheless, this study offers evidence of the impact of dietary diversity on children's nutritional status in the Musina Municipality of the Vhembe District, with its predominantly rural setting.

Despite these limitations, the research findings add to the body of literature that addresses the issue of dietary diversity and nutritional status in rural Sub-Saharan Africa and the Southern African Development Community (SADC) regions. It reports on the associations between dietary diversity and unemployment, household income, household water access and sanitation. Previously nutritional outcomes have been the main indicator of the water, sanitation and hygiene initiatives for low- and middle-income countries. Intermediary outcomes such as dietary diversity have received less attention^(65)^.

## Conclusion

Inadequate dietary diversity in young children in the rural settings in the Musina Municipality of South Africa is a significant concern. A lack of a diversified diet prevents children from obtaining enough of the nutrients needed for optimal development. This situation may be a main contributor to the high prevalence of underweight and stunting in this group of children. Parents/caregivers must be encouraged to include indigenous and locally available foods into their children's diet in order to increase the variety of nutrients. Local, affordable food options should be publicised to provide examples of a diverse diet. Additionally, pre-school menus should offer a variety of local and healthier foods to meet a child's nutritional and energy demands. The provision of information about good nutrition and planning and implementation of optimal food services in ECD centres could help to improve nutritional intake and long-term health in these children. Ideally, the South African government should increase the amount of each pre-schooler's subsidy for food and education, in accordance with the rate of inflation. Future nutrition education interventions should provide a stronger emphasis on consuming diverse and new foods from multiple food sources, in order to enhance optimal growth and development.
